# Transcriptomic Changes of Drought-Tolerant and Sensitive Banana Cultivars Exposed to Drought Stress

**DOI:** 10.3389/fpls.2016.01609

**Published:** 2016-11-04

**Authors:** Muthusamy Muthusamy, Subbaraya Uma, Suthanthiram Backiyarani, Marimuthu Somasundaram Saraswathi, Arumugam Chandrasekar

**Affiliations:** Crop Improvement Division, Indian Council of Agricultural Research-National Research Centre for BananaTiruchirappalli, India

**Keywords:** drought tolerance, banana transcriptome, RNA seq, wax synthase, osmo-protectants, Hsf, WRKY57, mRNA degradation

## Abstract

In banana, drought responsive gene expression profiles of drought-tolerant and sensitive genotypes remain largely unexplored. In this research, the transcriptome of drought-tolerant banana cultivar (Saba, ABB genome) and sensitive cultivar (Grand Naine, AAA genome) was monitored using mRNA-Seq under control and drought stress condition. A total of 162.36 million reads from tolerant and 126.58 million reads from sensitive libraries were produced and mapped onto the *Musa acuminata* genome sequence and assembled into 23,096 and 23,079 unigenes. Differential gene expression between two conditions (control and drought) showed that at least 2268 and 2963 statistically significant, functionally known, non-redundant differentially expressed genes (DEGs) from tolerant and sensitive libraries. Drought has up-regulated 991 and 1378 DEGs and down-regulated 1104 and 1585 DEGs respectively in tolerant and sensitive libraries. Among DEGs, 15.9% are coding for transcription factors (TFs) comprising 46 families and 9.5% of DEGs are constituted by protein kinases from 82 families. Most enriched DEGs are mainly involved in protein modifications, lipid metabolism, alkaloid biosynthesis, carbohydrate degradation, glycan metabolism, and biosynthesis of amino acid, cofactor, nucleotide-sugar, hormone, terpenoids and other secondary metabolites. Several, specific genotype-dependent gene expression pattern was observed for drought stress in both cultivars. A subset of 9 DEGs was confirmed using quantitative reverse transcription-PCR. These results will provide necessary information for developing drought-resilient banana plants.

## Introduction

Drought is one of the limiting environmental factors that affect crop production. According to Rabara et al. (2015) drought stress affects 64% of the global land area and expected to cause significant yield loss in crop plants. Bananas (perennial monocotyledonous herbs of the order Zingiberales, a *Musa* spp.), including dessert and cooking types, are vital for food security in many tropical and subtropical countries. Drought stress causes significant yield loss in bananas which are famously known as a commercial crop of the tropical and subtropical regions of the world. Requirement of an annual rainfall of 2000–2500 mm (evenly distributed) throughout the year is necessary for banana production. As high as 65% yield loss were reported in rainfed banana production areas to drought stress (Van Asten et al., 2010). Most cultivated banana varieties are parthenocarpic and mostly triploids with AAA, AAB, and ABB genome constitution. Banana varieties with AAB or ABB genome constitution are said to be more drought tolerant and hardy due to the presence of the B genome (Davey et al., 2009; Vanhove et al., 2012). Therefore understanding the molecular basis of how banana plants with different genomic groups respond to drought/water deficit stress is a key for developing drought tolerant banana plants.

Drought responses are notoriously multigenic and quantitative with strong environmental effects on phenotypes. Generation and analysis of expressed sequence tags (ESTs)/cDNAs has been successfully used for long to identify novel genes and their expression levels in specific tissues/species/stress conditions in broad range of model as well as non-model organisms. With the development of high-throughput DNA sequencing technologies, next generation sequencing (NGS) methods were extensively used to obtain a depth of sequencing that is sufficient to cover the transcriptome of an organism many fold and allow quantification of the detected transcripts (Yates et al., 2014). Application of NGS in cereals, legumes, and fruits etc. produced large amounts of EST data that have been submitted to various DNA databases (Lu et al., 2012; Thumma et al., 2012; Ding et al., 2014). The NGS technologies provide a cost-effective means of sequencing the transcriptome of higher plants like banana. Transcriptome profiling provides insights into important regulatory genes like transcription factors (TFs), kinases and other stress induced gene expressions that are involved in plant response to stress. To date, there are no reports on high-throughput sequencing based comparative transcriptome profiling for drought tolerance in banana.

Plant adaptation to drought is dependent on molecular networks for drought perception, signal transduction, expression of a subset of genes and production of metabolites that protect and maintain the structure of cellular components (Fleury et al., 2010). Stress responsive genes can be classified into types, ‘regulatory proteins’ like protein kinases and TFs which are come to play a role in early stress and ‘effector proteins’ which are responsible for several modifications in plant cells to mitigate stress effects (Kalyna et al., 2011; Tripathi et al., 2014). TFs are major players in drought stress signaling and TFs generally constitute major portion of transcriptionally active regions in banana genome (D’Hont et al., 2012) and expected to act as hub for signaling webs. The main TFs in this network include MYB, bHLH, bZIP, ERF, NAC, and WRKY etc. Moreover, overexpression of some of drought stress responsive TFs have been shown to confer drought tolerance (Hong et al., 2016; Yu et al., 2016). At the cellular level, plants respond to drought with changes in gene expression which lead to several other modifications at transcriptional and translational modifications, accumulation of osmo-protectants, enhanced water uptake (e.g., Aquaporin), reduction in transpirational water loss, strong antioxidants, heat shock proteins (hsps) and other stress tolerance proteins like dehydrin, metabolite abundances etc. (Phung et al., 2011; Wan et al., 2011; Hossain et al., 2015; Pandey and Shukla, 2015). Production of biologically functional components and metabolites considered to be essential for maintaining key cellular/metabolic process in plant cells under stress (Krasensky and Jonak, 2012). Plant cells known do osmotic adjustment by accumulation of osmolytes such as sugars (raffinose, trehalose, and stachyose), sugar alcohols, and proline which in turn enhance drought stress tolerance (Kishor et al., 2005; Kang et al., 2011; Wan et al., 2011; Sengupta et al., 2015; Liu et al., 2016). Drought stress induced expression of trehalose-6-phosphate synthase (TPS) leads to trehalose accumulation in banana expected to improve drought tolerance (Davey et al., 2009; Fernandez-Marin et al., 2010).

Abscisic acid (ABA) in drought stress has been shown to play crucial roles in regulating the drought response, and its metabolic pathway involves multiple steps and genes, generally known as stress hormone (Savchenko et al., 2014; Guerra et al., 2015). ABA mediated stress signaling and downstream gene expression is carried out by ABA signaling complex comprised of ABA receptors [PYR/PYR-like, (PYL), RCAR], protein phosphatase 2C (PP2C), and sucrose non-fermenting 1-related protein kinase 2 (SnRK2). The drought induced ABA in turn activates a series of TFs to direct transcriptional reprogramming to withstand drought stress. Moreover, modifications in cell wall compositions and membrane stability are expected to critically control stress tolerance (Dong et al., 2011). In some cases, stress induced lignification too helps plants to strengthen cell wall and in turn prevents cell collapse (Lee et al., 2007; Yoshimura et al., 2008; Gall et al., 2015). Similarly, Protein ubiquitination (post translational modification) crucially participates in regulation of stress response and contribute to stress tolerance (Stone, 2014; Dametto et al., 2015). However, not all transcribed messenger RNA (mRNA) in stressed plants are destined to be translated; most of these mRNAs are either degraded (Anderson and Kedersha, 2006) or post-transcriptionally processed into small non-coding RNAs like siRNA, miRNA. Small RNAs like microRNA, siRNA were too reported to be differentially expressed to drought stress (Wang and Chang, 2011; Muthusamy et al., 2014). As like protein coding genes, overexpression of some of these microRNAs in plants enhanced stress tolerance.

Centuries of natural selection and breeding efforts in different agro-climatic conditions led to the evolution of banana cultivars/landraces with a wide range of abiotic stress tolerance including drought tolerance (Zhang et al., 2016). Banana cultivar, Saba (ABB) identified as drought-tolerant is an excellent genetic resources for stress tolerance. Under stress, cv. Saba produced relatively higher amount of epicuticular wax content, proline and free amino acids and produced relatively good yield than few other tolerant banana cultivars (Ravi et al., 2013; Surendar et al., 2013). On the other hand, banana cv. Grand Naine (AAA), a Cavendish clone, resulted into complete failure of crop under drought stress and designated as highly susceptible for drought stress used in this study. Ironically, banana cv. Grand Naine has long been known as widely exported banana variety across global markets. The comparative analysis of transcriptomic response of these genotypes would help to understand the molecular basis of drought tolerance and to accelerate development of drought-tolerant plants (Lenka et al., 2011). The differentially regulated genes and its networks, molecular genetic pathways to drought stress tolerance and other inputs will serve as useful resources for establishing functional role of the molecular events during drought stress response in banana.

## Materials and Methods

### Plant Growth and Treatments

Banana (*Musa* spp.) plants of two cultivars, the drought-tolerant, ‘Saba’ (ABB genome) and drought-sensitive, ‘Grand Naine’ (AAA genome) were used in the present study. The growth conditions, drought stress imposition, sample collection and cDNA library construction methods were followed according to Muthusamy et al. (2015). Cultivars each 50 uniform-sized (≈1.0 kg and 90 days old) are planted into cement pots (48 cm diameter, 38 cm depth, one plant per pot) and maintained at field conditions (temperature 39°C/27°C and humidity 40%/85% day/night) with optimum watering conditions for 60 days prior to treatments. Progressive soil moisture-deficit stress was imposed on a group of 25 plants of both cultivars simultaneously by withholding water for 24 days continuously; the other set of the plants were continued to watering and treated as controls. Leaf samples of each treatment were collected from three independent plants of controls and drought stressed plants of two cultivars simultaneously at different time intervals like 7,14, 20, and 24 days after stress and was frozen with liquid nitrogen and stored at -70°C until used for cDNA library constructions. The volumetric soil water content at the end of the drought condition was recorded at root zone (at the depth of 200 mm) for the stressed plants was 9.1 and 33.5% for the control plants using ML2X probe/HH2 Moisture meter (Delta-T Devices, Cambridge, Great Britain). The surface area of the soil water content at the depth of 50mm was recorded approximately as 4.8% for stressed and 30.6% for control plants.

### Construction of cDNA Library and Illumina Deep-Sequencing

According to Muthusamy et al. (2015), total RNA was isolated from control and drought stressed leaf samples (tolerant and sensitive) collected at four time intervals for each treatment and then pooled separately to prepare 4 cDNA libraries (CT-control tolerant; DT-drought stressed tolerant; CS- control sensitive; DS-drought stressed sensitive) using mRNA-Seq assay kit. Three biological replicates were used for each treatment in this study. The library construction was performed as per the Illumina TruSeq RNA library protocol outlined in “TruSeq RNA Sample Preparation Guide” (Part # 15008136; Rev. A; Nov 2010). A 4 gigabase in-depth sequencing of library was performed using an Illumina HiSeq^TM^ GAII Analyzer instrument (Genotypic Technology, Bangalore, India) to obtain more detailed information about gene expression. Each paired-end library had an insert size of 200–700 bp. The average read length of 90 bp was generated as raw data.

### Differential Gene Expression (DGE) Analysis

Reference genome based transcriptome assembly for clean reads was performed to generate non-redundant unigenes using Cuﬄinks v2.0.1 method. The direction of the resulting unigenes was identified by performing BLASTX searches against UniProt database. The expression levels of unigenes were measured as the number of clean reads mapped to its sequence. The number of clean reads mapped to each annotated unigene was calculated and then normalized to RPKM and adjusted by a normalized factor. TopHat2 was used to assemble transcripts for banana RNA-seq reads by mapping to reference genome, *Musa acuminata* and cuﬄinks to measure their relative abundances was applied. The summation of FPKM (fragments per kilo base of transcript per million mapped reads) values for every transcript associated with a particular gene gives the expression measurement, in FPKM (Trapnell et al., 2009). The DGE is calculated by cuffdiff program using the ratio of control vs. treated FPKM values for every gene (Reference). Genes with fold change (FC = log fold change to the base 2) >1, < -1 and ≤1 were considered as up, down and neutrally regulated.

### Quantitative-Reverse Transcription PCR

Total RNA was extracted from tolerant control (CT), drought-stressed tolerant (DT), sensitive-control (CS) and drought-stressed-sensitive (DS) leaf samples of three time points (7, 14, and 24 days after stress) using the Spectrum^TM^ Plant Total RNA kit (Sigma-Aldrich, St. Louis, MO, USA). Each reverse transcription reaction was performed in 20 μl using RevertAid First Strand cDNA synthesis kit (Fermentas, Hanover, MD, USA) with 2 μg of total RNA (DNase-treated) as template along with random hexamer primers. The reaction conditions for cDNA synthesis were followed as per manufacturer’s recommendations (60 cycles of 42°C for 30 s, 45°C for 30 s, 50°C for 1 s and then at 70°C for 5 min). A 0.5 μl aliquot of the cDNA reaction was then used as a template for amplification by LightCycler 480 (Roche) real-time PCR systems using gene-specific forward and reverse primers. Annealing temperature for each primer and all other information were presented in Supplementary Table 1. The relative transcript level was normalized with *Musa* 25S rRNA gene.

## Results

**FIGURE 1:**
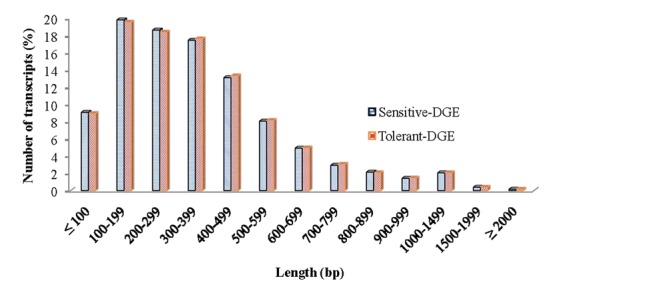
**Size distribution of the assembled contigs and unigenes from drought-tolerant and drought-sensitive banana libraries derived from RNA seq data**.

**FIGURE 2:**
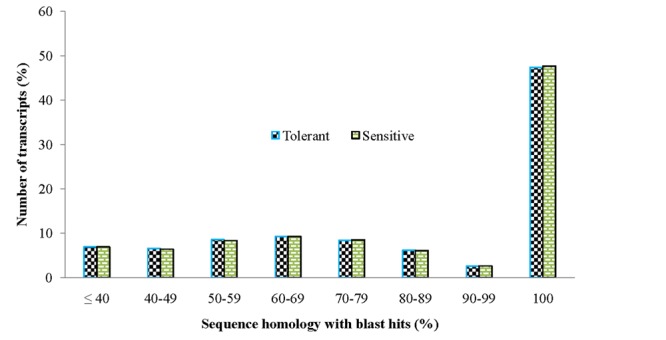
**Comparison of unigene sequence homology (in percentage) to blast hits/orthologous genes from viridiplantae groups**.

In order to achieve a broad survey of genes associated with drought stress response of drought-tolerant and sensitive banana cultivars, total RNA was extracted from control and drought stressed leaf tissues (pooled) from both cultivars. Using an Illumina paired-end sequencing platform, 162.36 million reads from tolerant (CT, DT) and 126.58 million reads from sensitive libraries (CS, DS) were produced and mapped onto the reference *Musa acuminata* genome sequence and assembled into 23,096 and 23,079 non-redundant unigenes (Supplementary Data 1). The sequence reads obtained in this study are available in the NCBI Sequence Read Archive (SRA) under accession number SRP087441. The size of the significant unigenes and blast hit distributions for the two DEG libraries across viridiplantae groups were almost consistent (**Figures 1** and **2**), which implied that the Illumina sequencing solution was reproducible and reliable. The length of assembled transcript sequences from RNA seq used for digital gene expression ranges from 33 to 12011 bp in tolerant and 33-7824 in sensitive cultivars in the present study (Supplementary Data 1 and 4). The sequence length of more than 60% of the reported DEGs/unigenes was ranges from 100 to 500 bp for tolerant and sensitive libraries (**Figure 1**). Of these annotated *Musa* transcripts, nearly 50% of unigenes had high nucleotide sequence similarity (100% identity) to already existing annotated databases of other crop plants (**Figure 2**). However, small fraction of annotated transcript (10%) sequences has relatively less homology (<49%) to other species. The nucleotide sequences of annotated transcripts showed different level of sequence homology to different plant species. High sequence homology were found to *Musa acuminata* (Wild) (1,37,850 contigs) followed by *Arabidopsis thaliana* (1,04,727 contigs), *Oryza sativa* subsp. *japonica* (23,546 contigs) and *Oryza sativa* subsp. *indica* (3,113 contigs).

**FIGURE 3:**
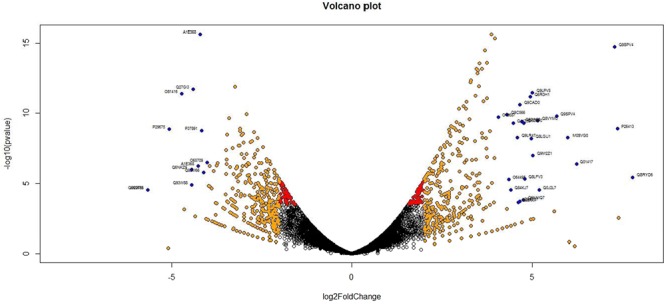
**Volcano plot indicates the significance of differentially expressed genes (DEGs) to drought stress from tolerant libraries**. Log fold changes vs. *p*-values were plotted using R programming language. Different color schemes represent DEGs of significance. Red (adjp ≤ 0.01), orange [adjp ≤ 0.01 and fold change = 2] and blue (adjp ≤ 0.01 and FC ≥ 4) represents gene expression to drought stress.

**FIGURE 4:**
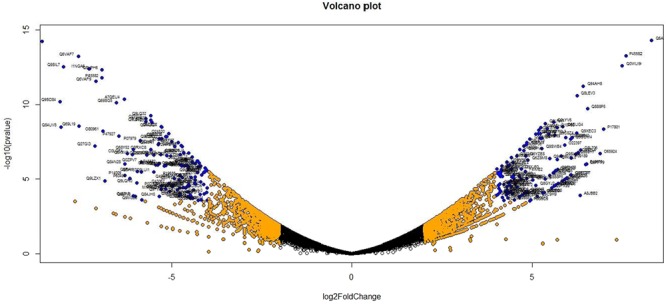
**Volcano plot indicates the significance of DEGs to drought stress from drought-sensitive libraries.** Log fold changes vs. *p*-values were plotted using R programming language. Different color schemes represent DEGs of significance. Red (adjp ≤ 0.01), orange [adjp ≤ 0.01 and FC = 2] and blue (adjp ≤ 0.01 and FC ≥ 4) represents gene expression to drought stress.

**FIGURE 5:**
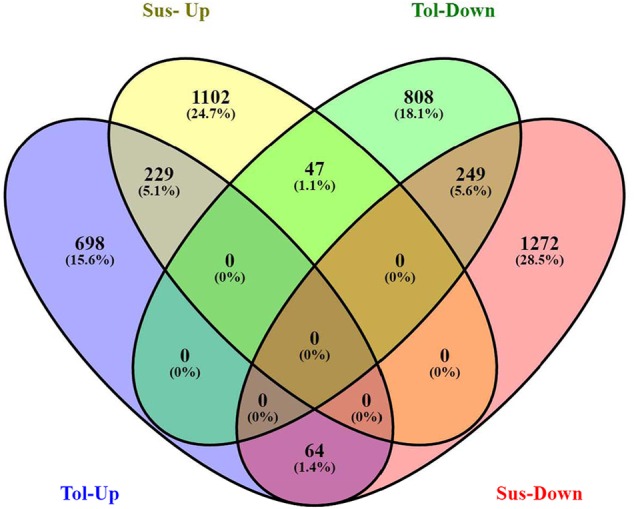
**Venn diagram represents the significantly overlapping unigenes between drought -tolerant banana cultivar Saba (ABB genome) and drought-sensitive banana cultivar Grand Naine (AAA genome)**.

Analysis of DGE between CT vs. DT and CS vs. DS libraries produced 8112 and 10537 significant DEGs (Supplementary Data 2). Annotation of DEGs with *Musa* gene database showed that 3597 and 4723 unigenes respectively from CT vs. DT and CS vs. DS are uncharacterized *Musa acuminata* genes. However, general annotation with UniprotKB of viridiplantae group showed only about 64 and 25 uncharacterized unigenes respectively. Further analysis showed that DGE between two conditions (control and drought) produced at least 2268 and 2963 statistically significant, functionally known, non-redundant DEGs respectively from tolerant and sensitive libraries. Of which, 991 and 1378 DEGs were up-regulated and 1104 and 1585 were down-regulated in CT vs. DT and CS vs. DS libraries respectively (Supplementary Data 3). The initial data for differential response of the contrasting cultivars under drought stress showed that 75.78% of the transcriptome of CT vs. DT libraries are unchanged while 46.25% of transcriptome were not regulated by drought stress from CS vs. DS libraries (Supplementary Table 2). The cluster and distribution of statistically important unigenes from CT vs. DT and CS vs. DS libraries were represented in different color schemes using volcano plot, where log fold change against p values were plotted (**Figures 3** and **4**). A total of 491 and 346 red colored spots [adjusted *p*-value (adjp) ≤0.01] were observed respectively in DT and DS. Similarly 381, 346 orange spots (adjp ≤ 0.01 and 2 fold change) and 55, 171 blue spots (adjp ≤ 0.01 and 4 fold change) were observed. The analysis of differentially expressed non-redundant unigenes of tolerant and sensitive genotypes showed that only about 13.2% were overlapping unigenes between them under stress (**Figure 5**). A total of 64 up-regulated DEGs of CT vs. DT found to be down-regulated in CS vs. DS libraries (**Figure 5**). Similarly among drought downregulated unigenes, 47 DEGs were enriched in CS vs. DS.

### Functional Classification of DEGs

Analysis of DEGs between the tolerant and sensitive libraries under stress should aid our understanding of the molecular events involved in drought stress response. To functionally classify those reported DEGs, International standardized gene functional classification system, i.e., Gene Ontology (GO) database was utilized.

**FIGURE 6:**
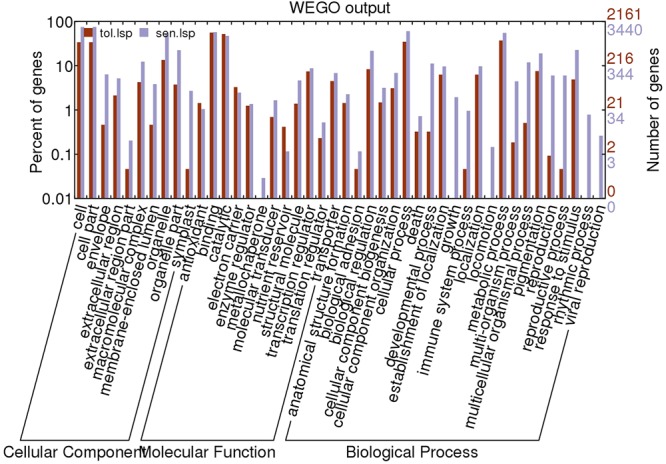
**Gene ontology classification of assembled unigenes**. The results are summarized into three main categories: biological processes, cellular components, and molecular functions. A total of 2161, 3440 statistically significant unigenes from drought-tolerant and sensitive banana libraries were assigned to GO terms.

Based on UniprotKB/Swiss-Prot annotations, 2160 (t); 3440(s) unigenes were assigned GO terms. Enrichment of Gene ontology terms using webGO tool to identify functional significance of DEGs was done for both CT vs. DT and CS vs. DS. In tolerant cultivars, under the biological process category (**Figure 6**), metabolic processes (37.1%) were the largest group, followed by cellular processes (Supplementary Table 3). However, cellular processes (60.5%) is topping in sensitive DEGs, followed by metabolic processes and response to stimulus (Supplementary Table 3). For cellular component and molecular function category, CT vs. DT and CS vs. DS were following uniform pattern. Under the cellular component category DEGs from CT vs. DT and CS vs. DS respectively showed that (**Figure 6**), unigenes were assigned to cell (33.9%; 75.02%), followed by organelle and membrane bound organelle. Similarly, for molecular function category (**Figure 6**), binding (56.01%; 57.2%) was the largest group, followed by catalytic activity, nucleic acid binding activity and transcription regulator activity respectively in CT vs. DT and CS vs. DS. Additional analysis with only upregulated genes revealed that DEGs associated with cellular components such as envelope, extracellular region, transcripts for molecular functions signal transducer, structural molecules and translational repressors were relatively overexpressed in DT than DS among 29 overrepresented groups. Among those groups, “response to abiotic stimulus” and “response to stimulus” under “biological process category accounts for 8 and 10% respectively” and expected to contain the most drought-related genes in this group.

### Over-Abundant or Most Represented Pathways

**FIGURE 7:**
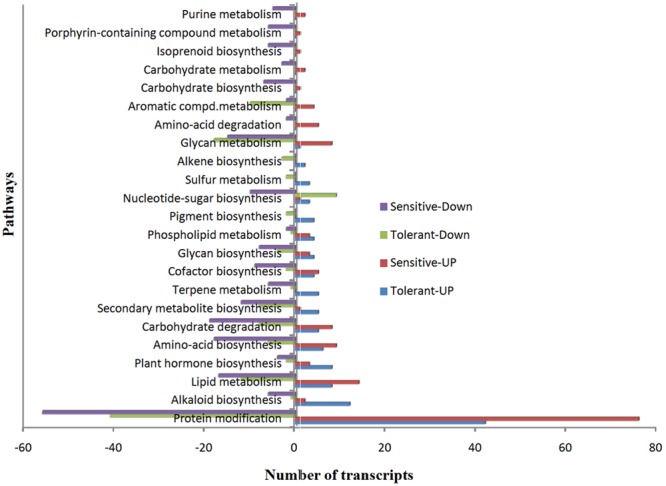
**The over-represented unigenes from differential gene expression data of tolerant and sensitive libraries and their KEGG terms**.

To understand the functions of DEGs, we mapped all non-redundant DEGs to terms in the KEGG database and found 41 and 44 pathways were enriched in CT vs. DT and CS vs. DS respectively. Furthermore, we found that most of the pathways (38, 71.11% – tolerant and 43, 66.6% – sensitive) were involved in various metabolic pathways, including lipid, carbohydrate, amino acid, glycan, cofactor, alkaloid, nucleotide-sugar, plant hormone, and other amino acids, terpenoids and biosynthesis of other secondary metabolites. However, protein modification is the single predominant pathway reported in both drought-tolerant (42-up-regulated, 41-down-regulated and 8- neutral) and sensitive banana cultivars (76-up-regulated, 56-down-regulated). Among metabolic pathways, lipid (8-up-regulated, 12-down-regulated, 3-neutral) and glycan metabolism (1-up-regulated, 18- down-regulated, 2- neutral) found to be predominant in tolerant libraries. Unlike DT, lipid metabolism (14- up-regulated, 17-down-regulated) followed by carbohydrate degradation (8-up-regulated, 19- down-regulated) found to be predominant in CS vs. DS. Among protein modifications, protein ubiquitination is the most significant category in both CT vs. DT and CS vs. DS libraries (**Figure 7**). DEGs of protein modification process were relatively highly induced in CS vs. DS than CT vs. DT. Apart from that, DEGs associated with plant hormone, secondary metabolite and alkaloid biosynthesis processes were also overexpressed in DT. While, DEGs of terpene metabolism and pigment biosynthesis were exclusively found in DT, carbohydrate degradation, amino acid biosynthesis, glycan metabolism and lipid metabolism are either largely reduced or exclusively found in downregulated DEGs of DS. Cellular localization of stress responsive proteins is also important to elucidate their function in plants. In the present study, the major variation in expression was observed for chloroplast associated transcripts, i.e., 60% of transcripts were regulated in DT. Interestingly, those transcripts were most highly reduced (77.47%) in DS which is 37% higher than DT. Moreover, DEGs associated with nucleus, cell membrane, secreted, peroxisome and vacuoles were found to be over-abundant in DS.

### Drought Responsive Transcription Factors

**FIGURE 8:**
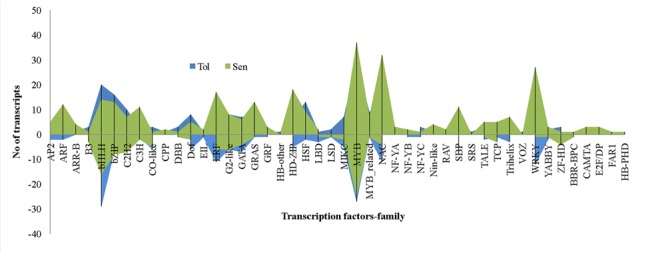
**Differentially expressed unigenes (DEGs) representing different families of transcription factors during drought stress**. In comparison, transcription factors from bHLH, bZIP, C2H2, DOF, HSF, and ZF-HD were overexpressed in tolerant libraries. Transcription factors such as ARF, ERF, MYB, WRKY, NAC etc. relatively overexpressed in sensitive libraries.

Transcriptional reprogramming is a central component of the response to drought stress. TFs are one of the important regulators at transcriptional level in higher plants like banana. In this study, 15.9% of total DEGs reported was coding for TFs. Of which, 222 and 302 TFs were induced in CT vs. DT and CS vs. DS respectively and the presence of 84 TFs were overlapped between both cultivars to drought stress. Moreover, expression of 21 TFs were found to be neutral under drought conditions in tolerant cultivars (DT). Around 46 TF families (classification as per CIRAD database for *Musa acuminata*) were found to be involved in *Musa* drought stress response which mostly represented by MYB, NAC, WRKY, bHLH, bZIP, ERF, and heat stress TFs (HSFs) (**Figure 8**). TFs such as HSF, bHLH, bZIP, MIKC, DOF, and C2H2 were found to be overexpressed in DT than DS. Our data showed that the number of up-regulated TFs was higher than down-regulated TFs in both DT (222-up, 172-down, 21-neutral) and DS (301-up, 114-down) and these were constituted by 41 and 43 TF families respectively. In DT, bHLH TFs were the main group of up-regulated genes (50 genes), followed by MYB, ERF, NAC, bZIP, WRKY, C2H2, HSF, DoF, G2-like, GATA, SBP, MIKC, HD-ZIP, ARF, GRAS, and MYB_related. The most highly induced TF gene was GSMUA_Achr8G07740_001 (7.7 fold) which encodes protein similar to *Arabidopsis* bZIP67 followed by (GSMUA_Achr8G01790_001) (5.76-fold), a homologue to AtWRKY26. MYB TFs were the main group of up-regulated genes (55 genes) in DS, followed by NAC, bHLH, WRKY, HD-ZIP, ERF, bZIP, C2H2, GRAS, SBP, C3H, ARF, and GATA.

### Musa Protein Kinases in Response to Drought Stress

Protein kinases (PKs) act as signal transducer/receptor proteins (esp. Receptor like PKs) at membrane and play a crucial role in phosphorylation events which activate and inactivate the downstream signaling cascades. Totally 220 and 276 PKs belonging to 82 PK (similar to *Musa acuminata* kinase) families were found to be differentially regulated in CT vs. DT and CS vs. DS. Mostly members from 10 PKs (RLK-Pelle_LRR-III, RLK-Pelle_LRR-XI-1, AGC_RSK-2 RLK-Pelle_DLSV, RLK-Pelle_RLCK-VIIa-2, RLK-Pelle_RLCK-V, CAMK_CDPK, CAMK_CAMKL-CHK1, STE_STE11 and NEK) were activated by drought. Altogether, 375 non-redundant PK members from both CT vs. DT and CS vs. DS were comprised from 151 different PKs. Class 1 PKs mostly represented by receptor-like kinases (RLK) followed by class-5 and 4 were largely induced in both cultivars (Supplementary Figure 1). Further, functional annotation revealed that 34 and 20 DEGs respectively from DS and DT were associated with drought, and salt stress as evidenced from functions of orthologous proteins.

### Differential Expression of Transcripts Associated with Biosynthesis of Various Metabolites

Biosynthesis of various components of plant cell has major impact on degree of stress tolerance. Altogether, 849 transcripts associated with biosynthesis process of variety of cellular components/metabolites including phytohormones, vitamins, fatty acids, free amino acids and free sugar, lignin and anticancer agents were identified from CT vs. DT and CS vs. DS. Of these, 357 (152 up-regulated, 175 down-regulated, 30-neutral) and 492 (153 up-regulated, 339 down-regulated) transcripts were identified from drought-tolerant (CT vs. DT) and sensitive banana genotypes (CS vs. DS). In comparison, approximately 69% of the transcripts involved in biosynthesis of different biomolecules were reduced in DS which is 20% more than DT. Only about 22% of genetic similarity (overlapped DEGs between cultivars) in biosynthetic transcripts was observed for drought stress. Transcripts associated with methionine, cellulose, L-ascorbic acid, UDP-D-xylose, UDP-glucuronate and nucleotide biosynthesis remained unchanged in DT. A total of 71 drought induced DEGs and 34 reduced DEGs were shown identical expression pattern for DT and DS. Drought stress regulated expression of hormones, ABA which is essential for signal transduction, cell wall or membrane components such as cellulose, homogalacturonan, lignin and wax and cell organelle components such as chlorophyll and GDP-mannose to drought conditions. In addition, DEGs involved in biosynthesis of fatty acids related processes such as synthesis of oxylipin, terpenoid/isoprenoids, isopentenyl diphosphate, 1-deoxy-D-xylulose 5-phosphate and dimethylallyl diphosphate and flavonoids were also regulated to drought stress. Drought stress seems mainly reduced biosynthesis of cellulose and fatty acids in both cultivars as evidenced from down-regulation of DEGs involved in these pathways in DT and DS. Among induced transcripts, predominant transcripts are from phytohormone metabolism related viz. ABA, cytokinin, brassinosteroid and gibberellins and jasmonic acids and their gene action followed by fatty acid and flavonoid biosynthesis related transcripts. The other notable induced transcripts are related with oligosaccharide synthesis pathways esp., transcripts involved in biosynthesis of trehalose, maltose, and raffinose and free amino acids synthesis viz. isoleucine, valine, L-serine, and proline.

### DEGs Associated with Osmotic Adjustments

Basically, plant cells can achieve osmotic adjustment by an accumulation of osmolytes (Wan et al., 2011). In this study, 3 DEGs associated with proline metabolism were identified. An increased level of Delta-1-pyrroline-5-carboxylate synthase (P5CS), an enzyme involved in proline biosynthesis, and decreased level of protein involving in proline transport activity named as Probable proline transporter 2 was observed in DS. While, Proline dehydrogenase-2 (mitochondrial), an enzyme responsible for conversion of proline into glutamate was enriched in DT to drought stress. Osmoprotective compounds include sugars, sugar alcohols, proline etc. are contributing to osmotic adjustment and thereby enhanced drought stress tolerance in plants (Liu et al., 2016). In the present study, significant changes in expression of the key enzyme coding genes involved in non-soluble sugar (starch) and soluble sugars (mannitol, trehalose, and sucrose) were observed to drought stress. Osmolytes include nitrogen-containing compounds such as spermidine forming enzymes, Polyamine oxidase 3 and Probable polyamine oxidase 4 were downregulated in DS. TPS8, TPS9 (chr10), TPS9 (chr3) and TPS6 encoding for trehalose synthesis were induced in DT while other isoforms such as TPS8 (chr1), TPPG and TPPA were downregulated. In DS, 4 isoforms of TPP6, TPPG along with TPS5 were observed, of which TPS5 and TPS6 (chr3) were only induced to stress. In addition stachyose (osmolyte) synthesizing enzymes was found to be upregulated in DS.

A larger increased relative abundance of enzymes such as Galactinol synthase 1 (chr8), Galactinol synthase 1 (chr11) and Galactinol synthase 4 involved in galactinol (osmolyte) biosynthesis was observed only in DT. The important glycolytic enzymes such Fructose-1,6-bisphosphatase and Fructose-bisphosphate aldolase were either remain unchanged to stress and or upregulated in DT while these enzymes were down-regulated in DS.

### Wax and Cell Wall Metabolism

Genes related to the cell wall are regulated by many different abiotic stresses, particularly water deficit stress (Gall et al., 2015). In this study, 60 of 71 and 43 of 57 cell wall metabolism associated DEGs from CT vs. DT and CS vs. DS were down-regulated. These DEGs are mostly representing cell wall enzymes such as β-glucosidase, cellulose synthase, expansin, extensin, glycosyl transferase, pectin esterase and xylosidase. *Musa* DEG homologous to At-XTH9 was overexpressed to fivefold in DT. Drought also induced XTH9 in DS along with XTH22, 27 and XTH 30 genes. Interestingly several of XTH proteins were downregulated in DT. DEGs representing EXPA4, EXPA7, EXPA8, EXPB15, EXLA1, and EXLA2 were induced in DT while EXPA4 was alone induced in DS. DEGs representing PAL (chr1) involved in lignin biosynthesis was induced in DT while, Caffeoyl-CoA *O*-methyltransferase (chr9) was induced in DS. Changes in the composition of wax due to stress must also be analyzed, as these features expected to increase membrane stability index in banana plants under stress (Surendar et al., 2013). In comparison to DS, 2 wax biosynthetic transcripts cuticular protein-1 (CUT1) and Long chain acyl-CoA synthetase 2 (LACS2) were overexpressed to 1.7977E+308 and 3.70449 folds respectively in DT. Apart from that, transcripts representing wax synthase was exclusively reported in DT and upregulated 1.4 fold to drought stress.

In our study, mRNA degrading enzyme such as 5′-3′ exoribonuclease (XRN) 2, 3, 4 were induced in DS might succumb the RNA pool during stress. In DT, a protein component of stress granule such as Argonaute- 1A, 1B, 1D and PNH1 were induced. In addition to that, few more members such as Argonaute 4A, 4B, Mel, PNH1, 10 and 12 were also induced in DS. Moreover, the initial analysis of contigs from RNA seq of DT and DS showed that drought has reduced number of contigs to larger extent in DS than their equivalent control, CT and CS.

Additionally, the qRT-PCR for randomly selected 9 DEGs (Supplementary Table 1) was tested to check the expression kinetics to those obtained from the RNA-Seq analysis. This independent experimental validation using qRT-PCR was carried out with cDNA synthesized from leaf samples separately from 7, 14 and 24 days of stress. The comparative transcriptome analysis between both cultivars suggested that following genes were overexpressed mostly in DT. Relative quantification of ABC transporter I family class 17 (Supplementary Figure 2A) (ABCI17) (Chr 10, 22689004–22692229 bp), Glutathione-*S*-transferase-parA (GST-ParA) (Chr 10, 18439813–18441155 bp) (Supplementary Figure 2B), NAC-8 (Chr 9, 1459292–1462947 bp) (Supplementary Figure 2C), Aquaporin (TIP3-1) (Chr 2, 13693837–13694758 bp) (Supplementary Figure 2D), Mannan endo-1, 4-beta mannosidase 7 (Chr 4, 2391414–2393463 bp) (Supplementary Figure 2E), HSP (22.3 kDa heat shock protein) (Chr 8, 2480371–2481396 bp) (Supplementary Figure 2F), ABA 8′-hydroxylase 3 (Chr 3, 4688557–4690876 bp) (Supplementary Figure 2G), and Phosphatase 2 C -13 (Chr 10, 22705003–22709616 bp) (Supplementary Figure 2H) showed more or less similar expression pattern to RNA seq data. The qPCR results showing higher/reduced expression in anyone point of drought or through cumulative expression of all three intervals (7, 14, and 24 days). Moreover, polyphenol oxidase (PPO) which showed no significant regulation to drought stress in our transcriptomics study (DT, DS) was also tested with qPCR. QPCR results showed that stress induced expression of PPOs in banana cultivars and relatively higher expression was noted in DS (Supplementary Figure 2I). Different algorithms, primer specificity and normalization factors involved in qPCR and RNA seq data would one of the several reasons for difference in expression pattern for PPOs.

### Identification of Drought Induced Unique DEGs from Tolerant Cultivars

To understand drought tolerance mechanism of banana cv. Saba, it is important to study unique gene expression profile during drought stress. Among DEGs (based on FPKM and *p*-values), 158 unique DEGs (Supplementary Data 2) comprised of 17 TFs and 47 enzymes were exclusively found to be enriched in DT. Of 158 DEGs, 6 DEGs encoding for ABA 8′-hydroxylase 1, Probable trehalose-phosphate phosphatase 6, Probable WRKY- 57, 17.5 kDa class I heat shock protein, Glutathione *S*-transferase F8, Ethylene-responsive TF ERF012, and ATP-dependent DNA helicase Q-like 5 were already known for their stress association in plants. Annotation of 139 DEGs failed to find best matched proteins with UniProtKB protein databases hence their functional association with drought stress is not clear.

## Discussion

Most of the nucleotide sequences reported in the present study were found to have high sequence homology to wild *Musa acuminata* reaffirming that almost all edible Musa clones are derivatives of most common ancestral banana genome (*Musa acuminata*). More number of unigenes was either up or down-regulated in DS than DT indicating sensitive genotypes were relatively hyper-responsive to stress and or easily disturbed by drought stress at molecular level. Our findings supports the reports of Yates et al. (2014) and Fracasso et al. (2016) who observed drought-sensitive genotypes of other plants (Clover and Sorghum) were found to be hyper-responsive to drought stress in comparison to their respective tolerant genotypes. This may be an indicative of absence of some transient and or primary layer of stress avoidance mechanism in sensitive cultivars to mitigate drought effects. Further, the quantum of drought reduced gene expression is larger than stress induced gene expression in DS. The larger reduction in gene expression of DS suspected either to be results of direct negative impact of stress or it could be part of stress adaptation strategy. The comparison of DEGs between CT vs. DT and CS vs. DS libraries clearly highlights that much portion of transcriptomic response to drought stress in tolerant cultivar is almost static (Supplementary Table S2). Importance should be given to these non-responsive transcripts of tolerant plants, since these transcripts could be the critical deciding factors of drought tolerance.

Gene Ontology term analysis showed that protein ubiquitination is the most significant category from CT vs. DT and CS vs. DS and ubiquitination event was highly induced in DS than DT (**Figure 7**) under drought conditions. Ubiquitination of proteins in plants are generally known as selective proteome degradation machinery to scavenge proteins of no more important for cell activities. However, the impact of ubiquitination on stress responses would depend upon substrate proteins (Stone, 2014). In DS, hyper response to stress hormone ABA, can be linked with high ubiquitination events because ubiquitination can be directly regulated by ABA under stress conditions. However, this process is also expected to provide proteome plasticity to plants. Although role of drought induced protein ubiquitination in DS at this juncture is not clear, a recent report had claimed that ubiquitination is a potential means for developing abiotic stress tolerant plants as reported in rice (Dametto et al., 2015). Therefore, whether protein ubiquitination is mere drought induced protein degradation or it is an adoptive response is yet to be known and warrants further in-depth study at least in banana plants. In our study, DEGs associated with plant hormone, cofactor synthesis and alkaloid biosynthesis was exclusively found in DT. This supports the findings of Selmar and Kleinwächter (2013) who reported drought mediated accumulation of alkaloid content and other secondary metabolites. Moreover, photosynthesis related transcripts were most highly reduced (77.47%) in DS which is 37% higher than DT suggesting that photosynthesis related processes were severely affected in DS. Down-regulation of more genes associated with photosynthetic apparatus in sensitive genotypes than tolerant genotypes was also noted in red clover transcriptome against drought stress by Yates et al. (2014). However, this could vary with other plants since it depends multi-factors like stress sensitivity and plant species (Yates et al., 2014).

In comparison, drought stress induced higher quantum of TFs in DS than DT, further confirms that transcriptome of sensitive cultivars are more active during stress and possibly involved in transcriptional reprogramming events. However, greater extent of reduction of TFs was noted in DT is not clear and information such as identification of precise target genes and regulatory pathways and their consequences may be essential to further understanding. Very recently Fracasso et al. (2016) too found that TFs were largely reduced in tolerant sorghum genotypes than sensitive genotypes under drought stress conditions. Around 46 TF families including MYB, NAC, WRKY, bHLH, bZIP, ERF, and HSFs were differentially expressed in banana cultivars to drought stress (**Figure 8**). MYB is the largest TF family regulated by drought stress in both cultivars followed by bHLH and NAC respectively in DT and DS. These TFs might play crucial roles in stress response and our results were consistent with previous studies which indicated that AP2/EREBP, bHLH, bZIP, HD-ZIP, HSF, MYB, NAC, WRKY, and zinc-finger protein TFs had vital roles in the plant response to drought stress (Davey et al., 2009; Mizoi et al., 2012; Sakuraba et al., 2015; Singh and Laxmi, 2015). The heat stress could induce expression of HSFs to protect plants from adverse effects of heat stress (Guo et al., 2016). This could be the reasons for larger induction of HSFs in DT. The second most highly induced gene (GSMUA_Achr8G01790_001) (5.76-fold), from DT libraries encodes a protein similar to AtWRKY26 which plays role in heat stress and confers thermo tolerance (Li et al., 2011). DEGs homologues to AtWRKY33 & 42 were found to be most highly induced in DS are known water deficit stress responsive TFs. WRKY42 involved in negative regulation of transcription. Most of the other up-regulated TF families, such as WRKY, NAC, C2H2, and HSF, participate in drought stress response (Huang et al., 2008; Nishizawa et al., 2008; Sakuraba et al., 2015; Singh and Laxmi, 2015). Particularly, some classes of bHLH TFs were known to bind with CBF genes (DREB1 and 2) under stress conditions and their interaction will contribute to abiotic stress tolerance including drought tolerance (Sreenivasulu et al., 2007). The ABA dependent NAC family members were highly upregulated in DS than DT and expected to mediate ABA-dependent gene expression under drought stress (Sakuraba et al., 2015).

In addition to TFs, several PKs were also differentially expressed to drought stress in tolerant and sensitive cultivars especially Leucine-rich (LRR) repeat RLKs overrepresented class- 1 kinases. Some of the RLK members have been reported to directly control water stress signaling in *Arabidopsis* (Tanaka et al., 2012). Like TFs, PKs also largely showed induced expression in DS than DT indicating the existence of distinct stress transduction cascade pathways. The hyper-responsive regulatory mechanism is expected to cause excessive changes in gene expression pattern that in turn disturb key cellular processes of plant cell, while delayed or slow response to drought stress has been considered as adoptive response (Jarzyniak and Jasinski, 2014).

Identification of drought induced unigenes in tolerant cultivars is highly important for understanding the drought tolerance mechanisms and to utilize for developing drought tolerant varieties in banana. Most of these proteins were localized and possibly destined to function at cell membranes followed by nucleus suggesting these unique DEGs would play crucial role in signal perception and transcriptional activation during stress. A total of 158 unique DEGs including few known potential drought tolerance candidates/stress responsive DEGs were exclusively found in DT. This specific genotype-dependent genes / genetic variation would be highly resourceful for genetic manipulation programs aimed to improve important agronomic traits like drought tolerance. Similar specific genotype-dependent genes were also reported for rice (*Oryza sativa* L) in response to drought stress (Huang et al., 2014). Overexpression of an *Arabidopsis* homolog – WRKY57 shown to confer significant drought tolerance and further gene interaction analysis indicated that WRKY57 can directly bind with W-box of RD29A and NCED3 promoter sequences to induce those genes under stress (Jiang et al., 2012).

### Accumulation of Osmo-Protectants and Other Secondary Metabolites

The production of biologically functional components/metabolites is essential for maintaining key cellular and metabolic processes in plant cells under stress (Krasensky and Jonak, 2012; Liu et al., 2016). Approximately 69% of the DEGs involved in biosynthesis of any biomolecules were reduced in DS which is relatively higher than DT. Genetic similarity for expression of these biosynthetic transcripts between cultivars is significantly less. Few metabolites that are essential for cell housekeeping was observed only in DT. Cellular signification is one of the drought induced processes said to play role in drought tolerance. However, in our study most of the lignin biosynthetic DEGs were reduced in both DT and DS libraries. Earlier Alvarez et al. (2008) also showed drought reduced expression of lignin biosynthetic transcripts in maize under drought stress. However, our results on lignification processes were contradictory to the findings of Lee et al. (2007) and Yoshimura et al. (2008) who found that drought induced expression of lignin synthesis genes in watermelon and white clover plants respectively. It can be reasoned from the fact that drought effect on lignin content may also depends on other factors like tissue types, time and intensity of stress as suggested by Fan et al. (2006). TPS driven trehalose accumulation was observed in DT supporting the results of Davey et al. (2009) who first evidenced drought stress induced trehalose accumulation in a drought tolerant banana variety. Literature evidences revealed that trehalose accumulation in transgenic crops had enhanced drought tolerance (Fernandez-Marin et al., 2010).

Differentially expressed genes encoding for glutamate were enriched in DT, which originally found to help plants to recover from stress periods (Verslues and Sharma, 2010). This may be an indicative of tolerance efficiency of banana cultivars. Moreover, this conversion is essential to maintain cellular homeostasis (Servet et al., 2012) and also important for providing energy under depleting nutrients (Liang et al., 2013). In DS, proline expected to accumulate, as P5CS was shown to overexpressed to drought stress. Although evidences supports for positive effects on proline accumulation on enzyme and membrane integrity, osmotic adjustment, and free radical scavenging (Kishor et al., 2005), the actual role of proline in plant osmotolerance remain controversial. The challenge remains to answer questions of how proline contributes to plant stress tolerance. As stated by Liu et al. (2015), drought stresses significantly influence the expression of genes responsible for soluble sugars such as mannitol, trehalose and sucrose in this study. In addition, supporting evidences showed that drought stress also induced galactinol synthase in *Arabidopsis* and its overexpression on transgenic *Arabidopsis* improved drought tolerance (Taji et al., 2002). The over-expression of DEGs involved in osmolyte synthesis is expected to mitigate the effect of drought primed osmotic stress in plant cells. Therefore, accumulation of galactinol in DT could protect plants from oxidative damage induced by drought stress (Nishizawa et al., 2008). As explained by Liu et al. (2013), drought regulated down-regulation of sucrose synthase (SUS) expected to lead to the insufficient production of UDP-glucose thus in turn slow the activation of cell division process thereby helping plants to effectively utilize energy for stress acclimation process.

In the present study, most of the DEGs associated with cell wall enzymes such as β-glucosidase, cellulose synthase, expansin, extensin, glycosyl transferase, pectin esterase and xylosidase were downregulated to drought stress in banana cultivars. Our results are in contrast to Dong et al. (2011) who reported that abiotic stresses including drought stress would strongly up-regulate genes involved in cell wall modification. However, few splice variants were also found to be induced in both cultivars. Xyloglucan endo-β-transglucosylases/hydrolases (XET/XTHs), and expansins play a central role in modulating cell wall extensibility, which mediates cell enlargement and expansion (Gall et al., 2015). The role of EXP in stress tolerance is inconclusive as reports claimed that overexpression of EXP in different species resulted in different degrees of stress tolerance, thus warranting further research on this aspect (Gall et al., 2015). Several classes of XTHs were reduced in DT and the role of XTHs in drought stress remains unclear although overexpressions of specific classes of XTHs were shown to confer drought tolerance (Cho et al., 2006). However, drought induced wax synthase in DT would directly help plants to delay drought symptoms (Jarzyniak and Jasinski, 2014). Further, drought induced expression of genes responsible for cuticular wax production supports the findings of Surendar et al. (2013) who reported higher wax content in tolerant banana genotypes, ‘Saba’ under drought stress. The wax deposition in banana leaves helps to maintain high membrane stability index. It can be further confirmed by the presence of visible ashy like wax coat in leaves of drought tolerant plant, Saba which is absent in leaves of banana cv. Grand Naine.

Under stress, some of the mRNA undergo mRNA decay pathways (mostly in PBs) and mRNAs are degraded by a 5′-3′ Exoribonuclease (XRN) (Souret et al., 2004). In our study, this mRNA degrading enzyme is largely induced in DS. This corresponds to the initial analysis which showed that large number of contigs were missing in DS (6471, 12.8%) in comparison to CS which showed relatively larger reduction than DT (2478, 4.8%). The reduction of number of contigs in DS might be due to overexpressed XRN driven mRNA degradation mechanisms. In addition, relatively high number of existence of DEGs associated with SG process in DS is also expected to contribute for the loss. Similarly, very less reduction of contigs for drought stress in DT can be attributed to the absence of induced expression of XRN members and also existence of only few members of SG formation process.

In summary, our study provided several new insights into transcriptomic response of drought tolerant and sensitive banana cultivars. Both genotypes exhibited a diverse transcriptional response under normal and drought conditions. However, the major outcome of this study would be identification of molecular basis of relative drought tolerance mechanisms. Drought tolerant mechanism of banana cultivar, Saba was attributable to several factor including static response of 75% of global transcriptome to drought stress and the enhanced expression of DEGs associated with wax production and or membrane stabilization related [wax biosynthetic transcripts cuticular protein-1 (CUT1), Long chain acyl-CoA synthetase 2 (LACS2), and wax synthase], protein modifications, biosynthesis of hormone, cofactor and alkaloids, TFs such as HSF, bHLH, bZIP, MIKC, DOF, and C2H2, and accumulation of osmo-protectants through Proline dehydrogenase-2, TPS8, TPS9 (chr10), TPS9 (chr3) and TPS6 encoding for trehalose synthesis, Galactinol synthase 1 (chr8), Galactinol synthase 1 (chr11) and Galactinol synthase 4 etc. In addition, our observation also led to identification of less down regulation of biosynthetic and photosynthesis related transcripts, 158 unique DEGs which include promising drought tolerant candidate genes in DT. With supporting evidences from previous studies and from our study, it is expected that enhanced expression of these DEGs in cv. Saba under drought appears to be in good agreement with high drought tolerance of this genotype. In part, existence of XRN driven mRNA degradation is expected to be responsible for drought sensitivity in cv. Grand Naine. This mRNA-Seq study is also provided information on qualitative and quantitative differences between cultivars. Taken together, the results of the comparative transcriptome analysis led to the identification of specific genotype-dependent genes responsible for drought tolerance. Our study will be highly resourceful for future crop improvement programs like development of drought-resilient banana plants.

## Author Contributions

MM, SU, and SB designed the research; MM, SU, and SMS performed the experiments; AC performed sequence analysis and submission; MM and SU wrote the manuscript.

## Conflict of Interest Statement

The authors declare that the research was conducted in the absence of any commercial or financial relationships that could be construed as a potential conflict of interest.
