# Supplementary material for: Transcriptomic Changes of Drought-Tolerant and Sensitive Banana Cultivars Exposed to Drought Stress
Source: Front Plant Sci. 2016 Nov 4;7:1609. doi: 10.3389/fpls.2016.01609 (PMC5095140; doi:10.3389/fpls.2016.01609)
Supplement: Supplementary file 1 [file Table_1.DOCX]

Supplementary data 1 List of primers designed for Real time PCR analysis to validate the transcriptome data

| **S.No** | **Transcript sequences of *Musa* - Illumina Hi-seq** | **Primer** | **Sequence (5'->3')** | **Strand** | **Length** | **Tm** | **GC%** |
| --- | --- | --- | --- | --- | --- | --- | --- |
|  | GSMUA_Achr3T06970_001 Abscisic acid 8'-**hydroxylase 3** (3:4688557-4690876) | ABH FP | CAGCTACCATATGCGGCTGA | Plus | 20 | 59.97 | 55.00 |
|  |  | ABH RP | TGCATGGAAGGTTCTGGCTT | Minus | 20 | 59.89 | 50.00 |
|  | GSMUA_Achr7T19550_001 ABC transporter I family member 17 | ABC FP | CATCTTTGGCATGGGCTCAG | Plus | 20 | 59.26 | 55.00 |
|  |  | ABC RP | AGTATCATCTCGCCTGTCGC | Minus | 20 | 59.69 | 55.00 |
|  | GSMUA_Achr10T14440_001 Probable protein phosphatase 2C 13 | P2C FP | GTGAATCACAAGCCGCTGAAT | Plus | 21 | 59.53 | 47.62 |
|  |  | P2C RP | GCAGTCAAAGCCATAGAACCA | Minus | 21 | 58.56 | 47.62 |
|  | GSMUA_Achr6T27000_001 Putative NAC domain-containing protein 8 | NAC8 FP | CACTTGGAAGGCAAAACGGG | Plus | 20 | 59.97 | 55.00 |
|  |  | NAC8 RP | GGAAGAAGTGCCGAACCAGA | Minus | 20 | 59.97 | 55.00 |
|  | GSMUA_Achr9T26030_001 Probable glutathione S-transferase parA | GST FP | TCGAGACTTACGCTGGCTTC | Plus | 20 | 59.83 | 55.00 |
|  |  | GST RP | AACTCGAAGACCTTGTGGGG | Minus | 20 | 59.60 | 55.00 |
|  | GSMUA_Achr8T03630_001 22.3 kDa class VI heat shock protein | HSP FP | TCCGAGTACGTCCTGAAAGC | Plus | 20 | 59.48 | 55.00 |
|  |  | HSP RP | TCCAGTCTCTTGCATCCGTC | Minus | 20 | 59.47 | 55.00 |
|  | GSMUA_Achr7T22010_001 Mannan endo-1,4-beta-mannosidase 7 | MAN FP | AGATTGCAGCTGAACCCGAA | Plus | 20 | 59.96 | 50.00 |
|  |  | MAN RP | TAAACGTGAACGGAGGCGAA | Minus | 20 | 59.97 | 50.00 |
|  | GSMUA_Achr6T29370_001 Polyphenol oxidase, chloroplastic | PPO FP | AAGCATCCCCACCACCTTTT | Plus | 20 | 59.81 | 50.00 |
|  |  | PPO RP | CCTATTCCGACGAGGACGTG | Minus | 20 | 59.97 | 60.00 |
|  | (2:13693837-13694758)  Aquaporin (TIP3-1) | AQN FP | CCAGAAGCTTGTAGAGGGGA | Plus | 21 | 53.15 | 52.38 |
|  |  | AQN RP | GTGGCCATGAACTCTGCGA | Minus | 19 | 54.64 | 57.89 |
|  | Musa 25S rRNA (AY651067) | FP | ACATTGTCAGGTGGGGAGTT | Plus | 20 | 57.3 | 50.0 |
|  |  | RP | CCTTTTGTTCCACACGAGATT | Minus | 21 | 55.9 | 42.8 |

PCR temperature Profile for each primers mentioned above in table

**Primer 1**

Target gene: ABH

Initial denaturation - 95^0^ C / 3 min

30 cycles of

Denaturation- 94^0^ C / 30 sec

Annealing-52^0^ C / 30 sec

Extension- 72^0^ C/ 30sec and then

Final extension- 72^0^ C/ 3 min

**Primer 2**

Target gene: ABC transporter

Initial denaturation - 95^0^ C / 3 min

30 cycles of

Denaturation- 94^0^ C / 30 sec

Annealing-53^0^ C / 20 sec

Extension- 72^0^ C/ 30sec and then

Final extension- 72^0^ C/ 3 min

**Primer 3**

Target gene: Probable protein phosphatase

Initial denaturation - 95^0^ C / 3 min

30 cycles of

Denaturation- 94^0^ C / 30 sec

Annealing-52^0^ C / 20 sec

Extension- 72^0^ C/ 30sec and then

Final extension- 72^0^ C/ 3 min

**Primer 4**

Target gene: Putative NAC domain-containing protein 8

Initial denaturation - 95^0^ C / 3 min

30 cycles of

Denaturation- 94^0^ C / 30 sec

Annealing-53^0^ C / 20 sec

Extension- 72^0^ C/ 30sec and then

Final extension- 72^0^ C/ 3 min

**Primer 5**

Target gene: Probable glutathione S-transferase

Initial denaturation - 95^0^ C / 3 min

30 cycles of

Denaturation- 94^0^ C / 30 sec

Annealing-52^0^ C / 20 sec

Extension- 72^0^ C/ 30sec and then

Final extension- 72^0^ C/ 3 min

**Primer 6**

Target gene: 22.3 kDa class VI heat shock protein

Initial denaturation - 95^0^ C / 3 min

30 cycles of

Denaturation- 94^0^ C / 30 sec

Annealing-53^0^ C / 20 sec

Extension- 72^0^ C/ 30sec and then

Final extension- 72^0^ C/ 3 min

**Primer 7**

Target gene: Mannan endo-1,4-beta-mannosidase 7

Initial denaturation - 95^0^ C / 3 min

30 cycles of

Denaturation- 94^0^ C / 30 sec

Annealing-51^0^ C / 20 sec

Extension- 72^0^ C/ 30sec and then

Final extension- 72^0^ C/ 3 min

**Primer 8**

Target gene: Polyphenol oxidase, chloroplastic

Initial denaturation - 95^0^ C / 3 min

30 cycles of

Denaturation- 94^0^ C / 30 sec

Annealing-51^0^ C / 20 sec

Extension- 72^0^ C/ 30sec and then

Final extension- 72^0^ C/ 3 min

**Primer 9**

Target gene: Aquaporin

Initial denaturation - 95^0^ C / 3 min

30 cycles of

Denaturation- 94^0^ C / 30 sec

Annealing-55^0^ C / 20 sec

Extension- 72^0^ C/ 30sec and then

Final extension- 72^0^ C/ 3 min

**Primer 10**

Target gene:

Initial denaturation - 95^0^ C / 3 min

30 cycles of

Denaturation- 94^0^ C / 30 sec

Annealing-50^0^ C- 58^0^ C***** / 20 sec

Extension- 72^0^ C/ 30sec and then

Final extension- 72^0^ C/ 3 min

***-** any temperature between 50-58^0^ C is suitable

Q12:
