# Supplementary material for: Transcriptomic Changes of Drought-Tolerant and Sensitive Banana Cultivars Exposed to Drought Stress
Source: Front Plant Sci. 2016 Nov 4;7:1609. doi: 10.3389/fpls.2016.01609 (PMC5095140; doi:10.3389/fpls.2016.01609)
Supplement: Supplementary file 2 [file Table_2.DOCX]

Supplementary Table 2 Differential gene expression profile in drought-tolerant and sensitive cultivars during drought stress from RNA seq data (Raw data)

| Differential gene expression | CS vs. DS | CT vs. DT |
| --- | --- | --- |
| Total Transcripts | 49,097 | 44,171 |
| Up-regulated | 12,726 (25.92%) | 5,152 (11.66%) |
| Down-regulated | 13,661 (27.82%) | 5,543 (12.54%) |
| Neutral | 22,710 (46.25%) | 33,476 (75.78%) |

CS- Control Sensitive libraries

CT- Control Tolerant libraries

DS- drought stressed sensitive libraries

DT- drought stressed tolerant libraries
