# Supplementary material for: Transcriptomic Changes of Drought-Tolerant and Sensitive Banana Cultivars Exposed to Drought Stress
Source: Front Plant Sci. 2016 Nov 4;7:1609. doi: 10.3389/fpls.2016.01609 (PMC5095140; doi:10.3389/fpls.2016.01609)
Supplement: Supplementary file 3 [file Table_3.DOCX]

Supplementary Table 3: Classification of differentially expressed transcripts according to GO terms (gene ontology)

| Gene ontology terms | Drought tolerant | Drought sensitive |
| --- | --- | --- |
| **1.Biological process**  **i.** metabolic processes  ii. cellular processes  iii. biological regulation  iv. response to stimulus | 802, 37.1%  751, 34.8%  180, 8.3%  106, 4.9% | 1899, 55.2%  2080, 60.5%  747, 21.7%  781, 22.7% |
| **2. Cellular component**  i. Cell  ii. Organelle  iii. Membrane bound organelle | 733: 33.9%  292: 13.5%  236: 10.9% | 2581; 75.02%  1785; 51.9%  1631; 47.4% |
| **3. Molecular function**  i. binding  ii. catalytic activity  iii. nucleic acid binding activity  iv. transcription regulator activity | 1210; 56.01%  1122; 51.9%  416; 19.3%  161; 7.4% | 1968; 57.2%  1623; 47.1%  734; 21.3%  301; 8.8% |
