# Supplementary figures and images for: Transcriptomic Changes of Drought-Tolerant and Sensitive Banana Cultivars Exposed to Drought Stress
Source: Front Plant Sci. 2016 Nov 4;7:1609. doi: 10.3389/fpls.2016.01609 (PMC5095140; doi:10.3389/fpls.2016.01609)

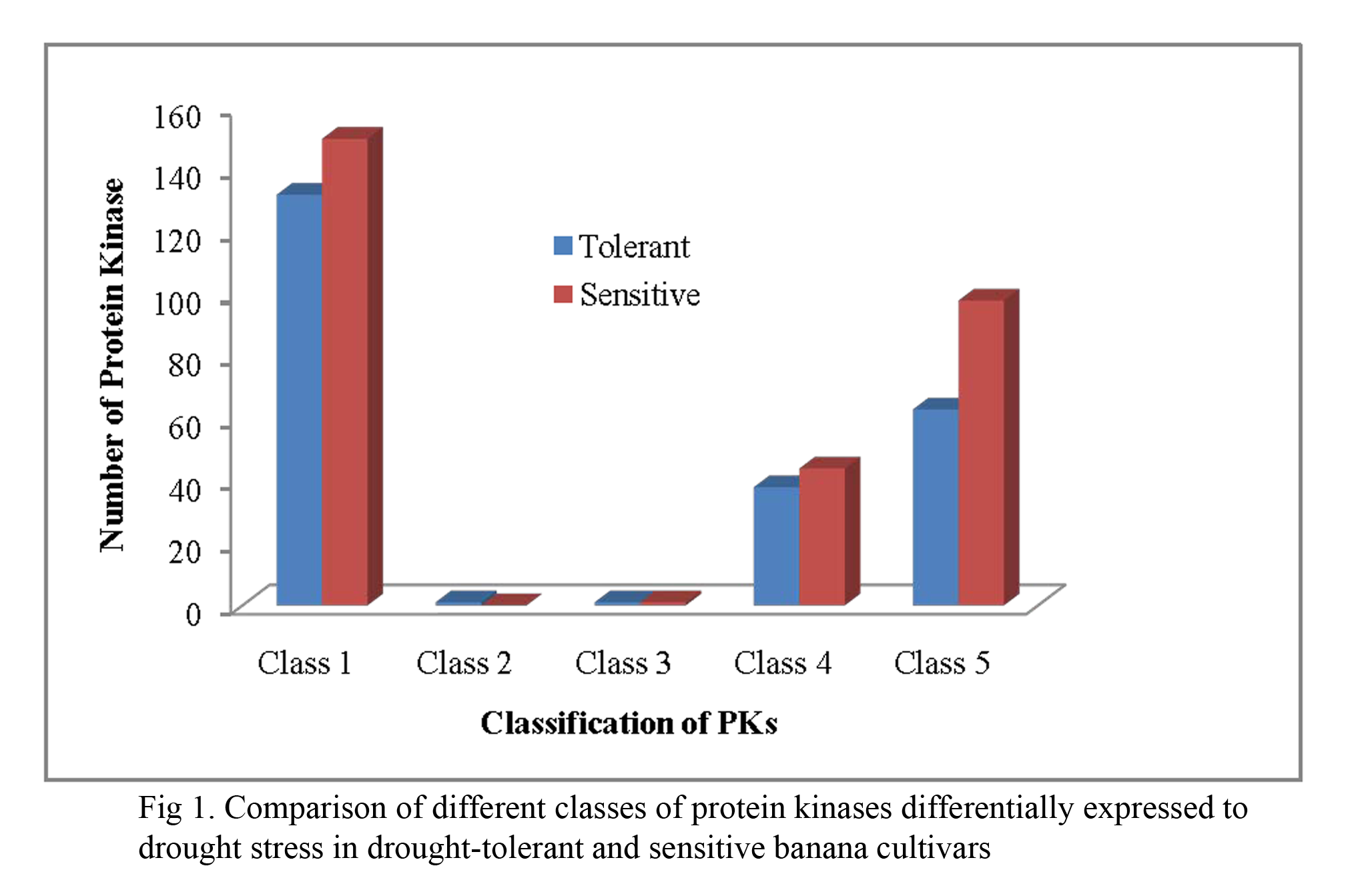

Supplement: Supplementary file 8 [file Image_1.TIF]

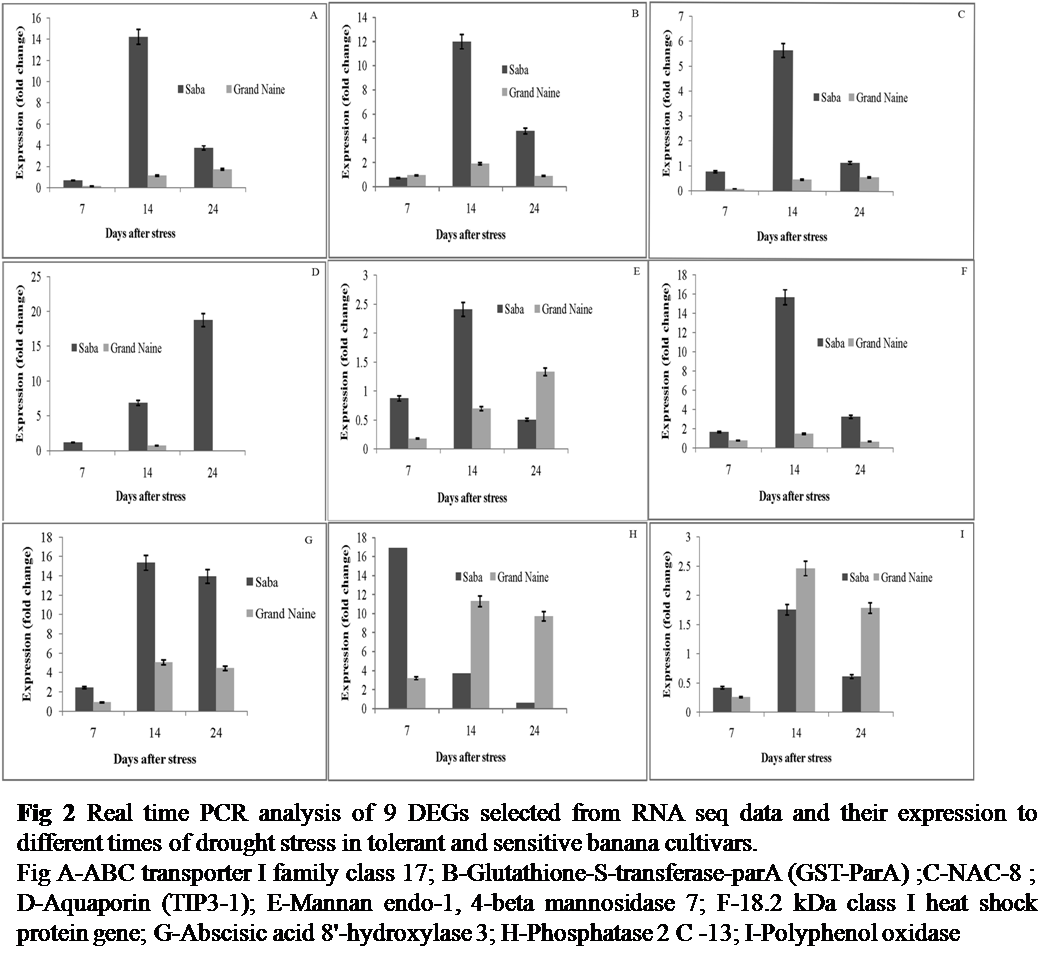

Supplement: Supplementary file 9 [file Image_2.TIF]
